# Assessment of Pulse Oximeter Perfusion Index in Pediatric Caudal Block under Basal Ketamine Anesthesia

**DOI:** 10.1155/2013/183493

**Published:** 2013-09-19

**Authors:** Zifeng Xu, Jianhai Zhang, Hao Shen, Jijian Zheng

**Affiliations:** Department of Anesthesiology, Shanghai First People's Hospital, Shanghai Jiaotong University, 650 Xin Songjiang Road, Shanghai 201620, China

## Abstract

Whether pulse oximeter perfusion index (PI) may be applied to detect the onset of caudal block in pediatric patients under ketamine intravenous basal anesthesia is investigated. 40 ASA I, 2–8-year-old boys scheduled for elective circumcision surgery were randomized into two groups. Group I: 20 patients were anesthetized by 2 mg*·*kg^−1^ ketamine intravenous injection (IV) followed by caudal block using 1 mL*·*kg^−1^ lidocaine (1%); Group II: 20 patients were anesthetized by 2 mg*·*kg^−1^ ketamine IV only. PI on the toe in Group II decreased by 33 ± 12%, 71 ± 9% and 65 ± 8% at 1 min, 15 min, and 30 min after ketamine injection. The maximum increase in MAP and HR after ketamine IV was 11 ± 6% at 3 min and 10 ± 6% at 2 min. Compared to the PI value before caudal injection of lidocaine, PI in Group I increased by 363 ± 318% and 778 ± 578% at 5 min and 20 min after caudal block, while no significant changes in MAP and HR were found compared to the baseline before caudal block. Thus, PI provides an earlier, more objective, and more sensitive indicator to assess the early onset of caudal block under basal ketamine anesthesia.

## 1. Introduction

Caudal block under basal ketamine anesthesia is widely used for pediatric lower abdominal and lower limbs surgeries in developing countries, especially for those uncooperative children. Successful caudal block can provide a safe, efficacious regional anesthesia and excellent postoperative pain control [1]. It may also provide the advantages in avoiding general anesthesia with trachea intubation, dramatically reducing the amount of ketamine used, therefore reducing the cardiovascular, psychological side effects of ketamine and finally fastening the recovery. However, the overall failure rate of caudal block in pediatric patients is about 4% secondary to the anatomical and developmental abnormalities of caudal canal [2, 3]. A reliable assessment of successful caudal block is critical for optimizing management of anesthesia in pediatric patients. The onset of caudal anesthesia is often assessed by pinpricking, cold stimuli, cutaneous temperature changes, or cremasteric reflex, and so forth, and it usually takes at least 15–30 min to confirm the efficacy of caudal block in children because of their unreliable response under deep sedation or general anesthesia [4]. Furthermore, these clinical signs and objective assessments do not act fast and reliable enough to provide a good feedback for anesthesiologists to optimize the anesthesia management.

Recent advances in pulse oximeter technology have expanded the abilities to measure more parameters, such as perfusion index (PI). Perfusion index is an assessment of the pulsatile strength at a monitoring site; it is calculated by means of pulse oximetry by expressing the pulsatile signal as a percentage of the nonpulsatile signal, both of which are derived from the amount of infrared light absorbed. PI can provide useful information about the peripheral perfusion status of the patients. Recent study suggested that PI could be used as an early and sensitive indicator to assess the development of epidural-induced sympathectomy in conscious adults; it is more sensitive than other parameters such as changes of skin temperature gradients or mean arterial pressure (MAP) [5]. Different from adult epidural anesthesia, pediatric caudal anesthesia is usually performed under ketamine anesthesia, and ketamine itself may change the sympathetic tone [6]; thus changes of PI in these patients may be unpredictable. Whether PI may also be applied to detect the onset of the pediatric caudal anesthesia under basal ketamine anesthesia remains to be elucidated. In this report, we compared the role of PI, MAP, heart rate (HR), and CR in detecting the onset of pediatric caudal block under basal ketamine anesthesia.

## 2. Patients and Methods

After the study protocol was approved by our institutional ethic committee and written informed consent from the parents was obtained, 40 ASA I, 2–8-year-old children scheduled for elective circumcision surgery were randomly allocated into two groups (20 patients, each group) using computer-generated random numbers. Group I: 20 patients were received caudal block under basal ketamine anesthesia; Group II: 20 patients under basal ketamine anesthesia only. All caudal blocks were performed by the same two anesthesiologists at left lateral decubitus position 5 min after 2 mg·kg^−1^ketamine IV injection. After loss of resistance of the needle and negative aspiration, a test dose of 2–4 mL lidocaine (1%) was injected. If no lump in the subcutaneous tissues, a feeling of resistance to the injection, or any systemic effects such as arrhythmias or hypotension occurred, then the remaining lidocaine was injected (total amount: 1 mL·kg^−1^) slowly. The injection speed should be less than 10 mL/30 seconds, and then the patients were immediately placed at supine position. Additional 0.5 mg·kg^−1^ ketamine IV bolus was given to the patients as needed during performing the caudal block.

All the patients were not given any premedication. HR and MAP were monitored by an S/5 anesthesia monitor (Datex-Ohmeda, Finland), and the PI was recorded using Masimo Radical-7 SET (Masimo Corporation, Irvine, CA). The pulse oximeter probe for monitoring the PI was placed on the left second toe and was wrapped in a towel to reduce heat loss and to avoid the light contamination from operating room. The CR was elicited by stroking the upper inner part of the thigh, and was judged to be present if the scrotum and testis on the examined side were pulled up by the contract of cremasteric muscle. The patients who have neuromuscular disease, cerebral palsy with or without mental retardation, and back sepsis, or whose CR were unable to be evoked before caudal block, were eliminated from the study. Group I: PI, HR, and MAP were recorded at 0, 5, 10, 15, and 20 min (T0, T5, T10, T15, and T20) following caudal drug administration. The CR was recorded as yes (Y) or no (N) at T0, T5, T10, T15, and T20. Group II: PI, HR, and MAP were assessed at 0, 1, 2, 3, 5, 10, 15, 20, 25, and 30 min following ketamine IV administration. The endpoints were expressed as an incremental change or as a relative change as stated below. The dPI in the toe was calculated as the absolute changes in PI following ketamine IV injection or lidocaine caudal administration with respect to baseline (T0), dPI = PI_IV  or  caudal_ − PI. The relative change in PI, the rPI, was expressed as the percentage changes from T0, rPI = dPI_caudal  or  IV_/PI_T0_ × 100. The changes of HR and MAP were also expressed as the changes in toe PI.

The effects of caudal administration of lidocaine under basal ketamine anesthesia and basal ketamine anesthesia itself on PI were assessed by repeated measures analysis of variance (RM-ANOVA). Drug effects at specific time (5, 10, 15, and 20 min and 1, 2, 3, 5, 10, 15, 20, 25, and 30 min) were determined using the simple repeated measures contrast option, which referenced the baseline values at T0; the conservative Greenhouse-Geisser modification was used if sphericity assumptions were not met.

For assessing the onset of adequate caudal block, we took the following criteria as indicators of onset of success of caudal block: absence of CR; a 100% increase of PI value from baseline; a 15% decrease of MAP from baseline; a 15% increase of HR from baseline. We recorded the number of patients who met or failed these criteria for each of these four parameters. ANOVAs test was used to compare the changes of these indices (PI, CR, MAP, and HR) over time. The ability of changes of PI value for assessing the onset of caudal block was compared separately with each of the other three parameters (CR, MAP, and HR) using McNemar's *χ* ^2^ test with a series of separate 2 × 2 contingency tables for each pair of tests at each time point. A *P* value ≤0.05 was defined as statistical significance. Statistical analysis was performed using SPSS version 13.0 (SPSS Inc., Chicago, IL).

## 3. Results

Forty-three children were initially recruited into the study, and three of them were excluded due to inability to identify caudal space or failure of caudal injection. Therefore, 40 children aged 2–8 years were randomized into two groups. There was no significant demographic difference between the two groups and no significant difference in baseline value of PI, MAP, HR, and CR at preinduction (Table 1).

**Figure 1:**
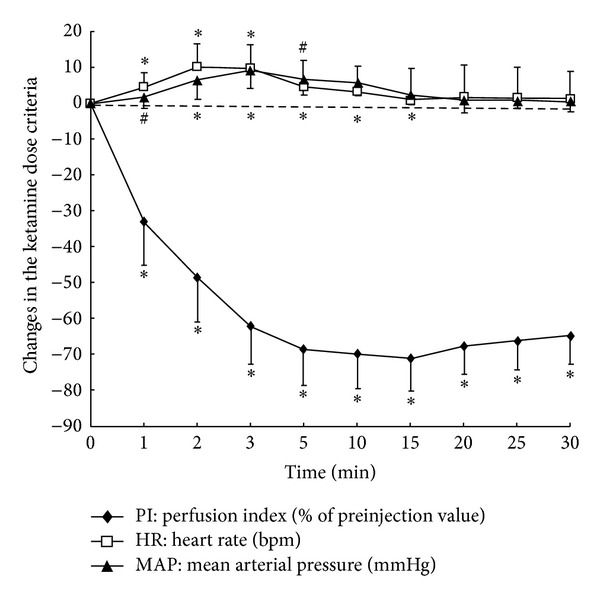
Changes in HR (bpm), MAP (mmHg), and percent perfusion index (PI) following intravenous ketamine administration. Indices were expressed as a change from T0 (preinjection values). Data were analyzed using repeated measures ANOVA, and statistical significance was defined as *P* ≤ 0.05. ^#^ *P* < 0.05 versus T0, and *P < 0.001 versus T0.

The changes of PI value on the toe, MAP, and HR following the caudal block under basal ketamine anesthesia were presented in Table 2. Following caudal administration of lidocaine, PI significantly increased by 2.01 ± 1.19 (representing a 363 ± 318% increase from T0) and 4.38 ± 1.86 (representing a 778 ± 578% increase from T0) at five and twenty minutes, respectively, after caudal block. There were no significant differences in MAP and HR following caudal lidocaine administration compared to their baseline values at T0. PI value on the toe significantly decreased, and MAP and HR markedly increased following ketamine IV injection itself (Group II). PI on the toe decreased to 1.58 ± 0.61 from 2.36 ± 0.79 (representing a 33 ± 12% decrease from T0) and to 0.66 ± 0.23 (the lowest value, representing a 71 ± 9% decrease from T0) at one and fifteen minutes following IV ketamine injection. PI began to slowly recover after T15, but significant reduction in PI still existed even at T30 (65 ± 8% decrease from T0). The maximum increase in MAP was 8.70 ± 5.22 mmHg (representing an 11 ± 6% increase from T0) at three minutes following IV ketamine injection, and the maximum increase in HR was 10.20 ± 6.28 bpm (representing a 10 ± 6% increase from T0) at two minutes following IV ketamine injection (Figure 1).

Following caudal administration of lidocaine, changes of PI value to meet the preset criteria for onset of successful caudal block were much earlier and more reliable than changes of other three indices in patients in Group I (Table 3). At five, fifteen minutes after caudal block, criteria of at least 100% increase of PI value from baseline were met in 17 of 20 patients and 20 of 20 patients, respectively. While at 5, 10, 15, and 20 minutes after caudal block, CR was intact in 20 of 20, 18 of 20, 11 of 20, and 0 of 20 patients, respectively, in Group I. However, following 15 min from caudal block, criteria of 15% decrease in MAP or 15% increase in HR from baseline were met in only 2 and 4 of 20 patients. Compared with the criteria of absence of CR and changes in MAP and HR for assessing onset of successful caudal block, criteria of changes of PI were met in most of the patients at 5 minutes, while none of the changes in MAP, HR, or absence of CR were met in all 20 patients. Therefore, it was very obvious that the PI was the superior index over other tests in assessing the effects of caudal block.

## 4. Discussion

Previous studies demonstrated that PI could provide an early and reliable indicator of the onset of epidural anesthesia and intravascular injection of epinephrine-containing epidural test dose in adults [5, 7]. However, different from epidural anesthesia or epidural testing dose in conscious adult patients, caudal blocks in pediatric patients were mostly performed under sedation or general anesthesia, such as ketamine or sevoflurane [8, 9]. Our data showed that ketamine itself can dramatically affect PI; whether PI can also provide an effective indicator in detecting the onset of caudal block in pediatric patients under ketamine anesthesia is still unknown.

Our study has shown that (1) ketamine IV injection in pediatric patients produced a fast and long-lasting decrease in peripheral PI; (2) caudal block not only reversed the decrease of PI on the toe caused by ketamine anesthesia in pediatric patients but also went far beyond the preinduction PI; (3) PI response criterion achieved 100% sensitivity and specificity in detecting the effects of caudal anesthesia under IV ketamine anesthesia in pediatric patients. On the other hand, neither HR nor MAP criteria were 100% reliable. Furthermore, the changes of PI caused by caudal block under ketamine anesthesia were much earlier than those of HR and MAP.

PI is a noninvasive numerical value of peripheral perfusion derived from calculating the amount of infrared light absorbed by pulsating arterial flow (AC) and nonpulsating blood and tissue (DC) by a pulse oximetry. The pulsating signal indexed against nonpulsating signal and expressed as ratio (AC⁡×100/DC%) is commonly referred to as the “perfusion index” [10–12]. Many factors such as blood volume, small peripheral vascular resistance, and elasticity of vascular wall may affect the changes of peripheral PI [13, 14]. Of these factors, peripheral vascular resistance which is regulated by autonomic nervous system contributes most to the changes of peripheral PI. It was reported that PI decreases caused by pain and other stressful stimuli were due to vasoconstriction of peripheral arterial bed rather than changes in the pulse pressure [15]; instead, PI increase on the toe following caudal block was most likely due to sympathectomy-related vasodilatation of peripheral arterial bed and redistribution of blood volume.

As what we speculated, ketamine as a widely used intravenous anesthetic in pediatric patients produced a quick and long-lasting decrease in peripheral PI due to its sympathomimetic effects via both central and peripheral mechanisms [16, 17]. Within one minute following ketamine intravenous injection, PI on the toe dropped from 2.36 ± 0.79 to 1.58 ± 0.61; by 30 min after ketamine injection, PI on the toe was 0.80 ± 0.26, still far below the baseline value of PI. The changes of MAP lasted about 15 minutes, and the changes of HR lasted about 5 min following ketamine injection. We stopped our observations at 30 min after intravenous injection of ketamine for avoiding excessive operation delay. Although ketamine increased MAP and HR within one minute, the changes ratios were much smaller than those of PI, and the time lasted much shorter compared to PI, suggesting that PI might be more sensitive in detecting the cardiovascular effects of ketamine. To our surprise, caudal block not only reversed the decrease of PI on the toe caused by ketamine anesthesia in pediatric patients, but also increased PI far beyond the preinduction PI value. Furthermore, the significant increases in PI had been shown at 2 min after caudal anesthesia (data not shown). However, there was no significant change in HR and MAP during the first 20 minutes after caudal block, suggesting that PI could provide an earlier, more reliable, and more sensitive indicator in detecting the onset of caudal block under ketamine anesthesia compared to MAP and HR.

We further compared the number of patients who meet predefined criteria for each of the bedside indices used for assessment of caudal block onset as Ginosar et al. did in their study [6]. Since PI was the major diagnostic tool for assessing efficacy of caudal block under investigation, we therefore defined a 100% increase in PI from the time of finishing caudal injection, a more rigorous criterion for PI compared to those for MAP or HR. In spite of this, PI is still able to detect onset of successful caudal block much earlier and more consistently than either MAP or HR. The drawback in our study was that we imposed arbitrary criteria on the endpoint being studied [5], although these endpoints were based on previous published data.

The disappearance of the cremasteric reflex has long been thought as a more reliable indicator of successful caudal block, but it only applies to male pediatric patients, and it usually takes much longer to show the effects of caudal block [18, 19]. In our study, we found that changes of PI on the toe in 85% of the children were more than 100% increase within 5 minutes of caudal block, but the CR in all the twenty patients tested was intact. By 15 minutes, only 45% patients' CR disappeared, although all patients' CR disappeared after 20 minutes following caudal block. Compared to CR, we further demonstrated that PI was an earlier and more objective indicator in detecting the early onset of caudal anesthesia under ketamine anesthesia, and it may apply to both male and female patients (data not shown). 

Skin temperature gradients have also been used as an effective indicator of sympathectomy [20–22]. We did not compare the changes of skin temperature with those of PI due to the lack of skin temperature probes. Therefore, further observation should be conducted to compare the effects of caudal block under ketamine anesthesia on PI and skin temperature.

Our data clearly demonstrated that an increase in PI is an early, reliable, and objective indicator of the successful onset of caudal anesthesia under ketamine anesthesia. Conversely, failure to increase in PI might give the anesthesiologist an early warning of failure of adequate caudal block, which may help the anesthesiologist to optimize the management of anesthesia and finally to avoid the side effects of ketamine or other adjunctive medicines overdose. 

## 5. Conclusion

PI provides an earlier, more objective, and more sensitive indicator to assess the early onset of caudal anesthesia under ketamine anesthesia. This result may encourage anesthesiologists to use PI instead of pinching, pinpricking, CR, and so forth, to assess the efficacy of pediatric caudal block under basal anesthesia, and to guide the management of pediatric caudal block.

## Figures and Tables

**Table 1:** Demographic data and baseline value in all patients.

	Group 1 (*n* = 20)	Group 2 (*n* = 20)	*P* value
Age (yr)	5.6 ± 1.9	5.3 ± 1.8	0.231
Height (cm)	108 ± 12	113 ± 11	0.182
Weight (kg)	25 ± 11	27 ± 9	0.534
Preinduction			
MAP (mmHg)	82.1 ± 11.3	80.6 ± 13.0	0.693
HR (bmp)	98.7 ± 16.5	100.2 ± 16.3	0.779
PI	2.3 ± 0.8	2.4 ± 1.5	0.788
CR (N/Y)	0/20	0/20	

MAP: mean artery pressure; HR: heart rate; PI: perfusion index; CR: cremasteric reflex; N: no; Y: yes.

Data are expressed as mean ± SD or numbers.

**Table 2:** Bedside indices for the onset of caudal block under ketamine basal anesthesia: changes over time following caudal lidocaine administration.

Time after caudal block (min)	PI toe	*P* value	dPI toe	MAP (mmHg)	*P* value	dMAP (mmHg)	HR (bpm)	*P* value	dHR (bpm)
0	0.70 ± 0.29	—	—	90.00 (14.19)	—	—	103.75 ± 18.05	—	—
5	2.70 ± 1.14	*P* < 0.001	2.01 ± 1.19	88.80 ± 13.80	*P* = 0.069	−1.20 ± 2.78	104.10 ± 18.87	*P* = 0.714	0.35 ± 4.21
10	3.56 ± 1.47	*P* < 0.001	2.87 ± 1.51	88.25 ± 13.37	*P* = 0.099	−1.75 ± 4.52	104.10 ± 20.02	*P* = 0.854	0.35 ± 8.39
15	4.67 ± 1.71	*P* < 0.001	3.97 ± 1.76	87.75 ± 13.56	*P* = 0.065	−2.25 ± 5.14	105.05 ± 21.70	*P* = 0.561	1.30 ± 9.81
20	5.08 ± 1.82	*P* < 0.001	4.38 ± 1.86	87.60 ± 13.31	*P* = 0.075	−2.40 ± 5.70	105.00 ± 21.39	*P* = 0.553	1.25 ± 9.27

PI: perfusion index; MAP: mean arterial pressure; HR: heart rate. Data are expressed as mean ± SD. *P* versus baseline (T0).

**Table 3:** Bedside indices for the onset of caudal block: numbers of patients meeting predefined “clinically obvious” targets indicative of onset of caudal block over time.

Pre-defined “clinically obvious” targets for positive test of onset of caudal block	Time after caudal injection (min)	Number (%) of patients reaching targets for positive test	Comparison with rPI for the same dose and time interval	
rPI toe (100% change from time 0)	5	17/20 (85%)	—	
	10	19/20 (95%)	—	
	15	20/20 (100%)*	—	
	20	20/20 (100%)*	—	
CR (absence %)	5	0/20 (0%)*	rPI > CR	0% for CR
	10	2/20 (10%)	rPI > CR	*P* < 0.001
	15	9/20 (45%)	rPI > CR	100% for dPI*
	20	20/20 (100%)	rPI = CR	100% for dPI, CR
rMAP (15% change from time 0)	5	0/20 (0%)*	rPI > rMAP	0% for rMAP
	10	1/20 (5%)	rPI > rMAP	*P* < 0.001
	15	2/20 (10%)	rPI > rMAP	100% for dPI*
	20	2/20 (10%)	rPI > rMAP	100% for dPI*
rHR (15% change from time 0)	5	0/20 (0%)*	rPI > rHR	0% for rHR
	10	3/20 (15%)	rPI > rHR	*P* < 0.001
	15	4/20 (20%)	rPI > rHR	100% for dPI*
	20	4/20 (20%)	rPI > rHR	100% for dPI*

PI: perfusion index; CR: cremasteric reflex; HR: heart rate; MAP: mean arterial pressure. Separate 2 × 2 contingency tables were constructed to compare rPI with CR (absence %), rPI with rMAP, and rPI with rHR at each time point. Values are number (ratio). *Some contingency tables were so one-sided that they could not be assessed using McNemar's test of symmetry, as there was either 100% positive data for rPI or 0% positive data for the comparison test. In these cases, the superiority of rPI was clearly self-evident.
